# VEGF Modulates Neurogenesis and Microvascular Remodeling in Epileptogenesis After Status Epilepticus in Immature Rats

**DOI:** 10.3389/fneur.2021.808568

**Published:** 2021-12-24

**Authors:** Wei Han, Li Jiang, Xiaojie Song, Tianyi Li, Hengsheng Chen, Li Cheng

**Affiliations:** Department of Neurology Children's Hospital of Chongqing Medical University, National Clinical Research Center for Child Health and Disorders, Ministry of Education Key Laboratory of Child Development and Disorders, China International Science and Technology Cooperation Base of Child Development and Critical Disorders, Chongqing Key Laboratory of Pediatrics, Chongqing, China

**Keywords:** VEGF, neurogenesis, microvascular remodeling, epileptogenesis, SE

## Abstract

Neurogenesis and angiogenesis are widely recognized to occur during epileptogenesis and important in brain development. Because vascular endothelial growth factor (VEGF) is a critical neurovascular target in neurological diseases, its effect on neurogenesis, microvascular remodeling and epileptogenesis in the immature brain after lithium-pilocarpine-induced status epilepticus (SE) was investigated. The dynamic changes in and the correlation between hippocampal neurogenesis and microvascular remodeling after SE and the influence of VEGF or SU5416 injection into the lateral ventricles at different stages after SE on neurogenesis and microvascular remodeling through regulation of VEGF expression were assessed by immunofluorescence and immunohistochemistry. Western blot analysis revealed that the VEGFR2 signaling pathway promotes phosphorylated ERK and phosphorylated AKT expression. The effects of VEGF expression regulation at different stages after SE on pathological changes in hippocampal structure and spontaneous recurrent seizures (SRS) were evaluated by Nissl staining and electroencephalography (EEG). The results showed that hippocampal neurogenesis after SE is related to microvascular regeneration. VEGF promotion in the acute period and inhibition in the latent period after SE alleviates loss of hippocampal neuron, abnormal vascular regeneration and inhibits neural stem cells (NSCs) ectopic migration, which may effectively alleviate SRS severity. Interfering with VEGF via the AKT and ERK pathways in different phases after SE may be a promising strategy for treating and preventing epilepsy in children.

## Introduction

Convulsive disorders are the most common emergency in pediatric neurology. The incidence of convulsions is higher in children than in adults (1), and children are more prone to severe convulsions and status epilepticus (SE), which is associated with more serious long-term nervous system sequelae such as epilepsy, cognitive impairment, and emotional disorders and thus greatly affects the quality of life of children (2–4). However, at present, the specific mechanism of epileptogenesis after SE is not fully understood, and there is no effective method for repairing convulsion-induced brain damage or effectively preventing epilepsy.

Epileptogenesis is a pathological process characterized by spontaneous recurrent seizures (SRS) that gradually emerge after SE. The progression from acute convulsive brain injury to epileptogenesis can be divided into the acute phase, latent phase and chronic phase according to the different physiological and pathological changes that occur in the brain and the occurrence of SRS. In animal model, 0–3 days after SE were identified acute period, 3–7 days after SE were identified the latency period and approximately after 7–10 days was chronic early stage. SRS appears from chronic early stage. Of course, the onset and duration of such changes are approximated (2). Studies have found that a series of molecular pathological changes occur in the brain during the latent phase (5) and that SE-induced brain damage in the immature brain is different from that in the mature brain. It may be possible to prevent epileptogenesis by identifying important targets related to the mechanism of epilepsy in the immature brain.

Previous research has widely recognized that SE increases neurogenesis both in animal models of epilepsy and human temporal lobe epilepsy (TLE) (6, 7). It is known that the immature brain has a greater regenerative capacity than the mature brain and SE-induced endogenous neurogenesis may compensate for hippocampal neuronal loss. However, our previous study revealed that hippocampal neurons in the immature brain undergo excessive apoptosis after SE and that endogenous neural stem cells (NSCs) in the subgranular zone (SGZ) of the hippocampal dentate gyrus (DG) exhibit a proliferative response and abnormal migration as in the mature brain (8). The abnormal migration of newborn granule cells leads to brain development disorders and increased susceptibility to convulsions through forming functional excitatory synapses in the molecular layer of the DG (MoDG) and excitatory recurrent circuits (9). SE-induced neurogenesis is a complicated process and may be regulated by numerous other factors. The microenvironment of NSCs is closely related to the proliferation, migration, differentiation, and survival of NSCs. Microvessels are some of the most important components of the NSCs microenvironment. In addition to providing nutritional support to NSCs, blood vessels affect the migration of NSCs in normal brains. Some studies have found that microvascular injury is characterized by blood-brain barrier (BBB) destruction and excessive angiogenesis after SE (10–14). Thus, we hypothesized that pathological angiogenesis after SE might affect the migration and integration of NSCs and thus cause neurons to migrate ectopically and glial cells to establish abnormal synaptic connections, form a pathological neural network, promote abnormal excitation of neurons, and participate in epileptogenesis (15). An increasing number of studies have confirmed that continuous proliferation of hippocampal NSCs and microvascular remodeling in pathological changes are involved in the pathogenesis of epilepsy, but the specific mechanism is still unclear (16–18).

Vascular endothelial growth factor (VEGF), as an important common factor between the nervous system and the vascular system, is a promising neurovascular molecular target (19); however, research on its role in epilepsy is limited. VEGF is a dimeric glycoprotein that was first isolated and purified from cultured bovine pituitary astrocytes in 1989 by Ferrara et al. (20). Its molecular weight is 34–45 kDa. VEGF is secreted by neural tube cells in the developing brain, and it mainly binds to its receptor to perform its biological functions. The members of the VEGF family that have been discovered thus far include VEGF-A, VEGF-B, VEGF-C, VEGF-D, VEGF-E, and placental growth factor. VEGF commonly refers to VEGF-A. The known VEGF receptors (VEGFRs) are VEGFR1 (Flt1), VEGFR2 (Flk-1), VEGFR3 (Flt-4), NP-1 and NP2 (21–23). The localization and functions of different VEGFRs are distinct. VEGF-A, which plays an important role in angiogenesis, endothelial cell proliferation and migration and the proliferation of NSCs, mainly exerts its biological functions by binding to VEGFR2 in the nervous system (24). Because VEGF has dual effects on blood vessels and nerves in the nervous system, some researchers believe that VEGF acts as a double-edged sword in epilepsy. It promotes the proliferation of blood vessels and contributes to the development of epilepsy. On the other hand, it inhibits hippocampal cell apoptosis and promotes the proliferation of NSCs, the survival of neurons, and the repair of damaged nerves (25–27). Previous research revealed that the dynamic expression of VEGF and VEGFR2 fluctuates during the development of epilepsy after SE, which is consistent with the trend in SRS (8, 28). VEGF can promote the regeneration of hippocampal NSCs in the acute phase after SE (8), suggesting that it may be involved in epileptogenesis. Does VEGF, in addition to promoting the proliferation of NSCs after SE, affect the regeneration of microvessels in the brain? Does VEGF have distinct effects in different stages of epilepsy development after SE? Does the temporal regulation of VEGF expression and receptor signaling pathways after SE have different effects and influence the development of epilepsy?

Since the proliferation of hippocampal NSCs after SE is not synchronized with vascular remodeling, we speculated that VEGF expression is regulated differently during different time windows after SE. Therefore, based on our previous studies, we performed the present study to investigate the critical role of VEGF and VEGFR-2 inhibitor SU5416 expression regulation and the time-dependent effect of the VEGF on neurogenesis and microvascular remodeling in the immature brain after SE to explore the role of VEGF in the development of epilepsy and to provide new ideas for the prevention and treatment of epilepsy.

## Materials and Methods

### Animals

Healthy male specific pathogen-free (SPF) Sprague-Dawley (SD) rats aged 18 and 20 days were provided by the Experimental Animal Center of Chongqing Medical University. All animal experiments were performed in accordance with protocols approved by the Animal Care Committee of Chongqing Medical University, Chongqing, China. The animals were housed in a controlled environment at a humidity of 60% and a temperature of 21 ± 1°C on a 12 h light/dark cycle (lights on from 7:00 AM−7:00 PM) and provided food and water ad libitum.

### Lithium Chloride-Pilocarpine-Induced Rat Model of SE

SE was induced in 20-d-old male SD rats. Seizures were scored as stages 1–5 based on Racine's five-point seizure scale (29). SE was induced *via* intraperitoneal (i.p.) injection of pilocarpine (30 mg/kg, Sigma) 18–20 h after lithium chloride injection (127 mg/kg, i.p., Solarbio, Beijing, China). Atropine (Shuanghe, Beijing, China) was administered via i.p. injection (1 mg/kg) 30 min after the onset of seizure. Sixty min after SE, all rats received a single dose of diazepam (10 mg/kg, i.p.). Rats that did not display SE and those that died during SE were excluded. Control rats were treated with the same volumes of saline.

**Figure 1:**
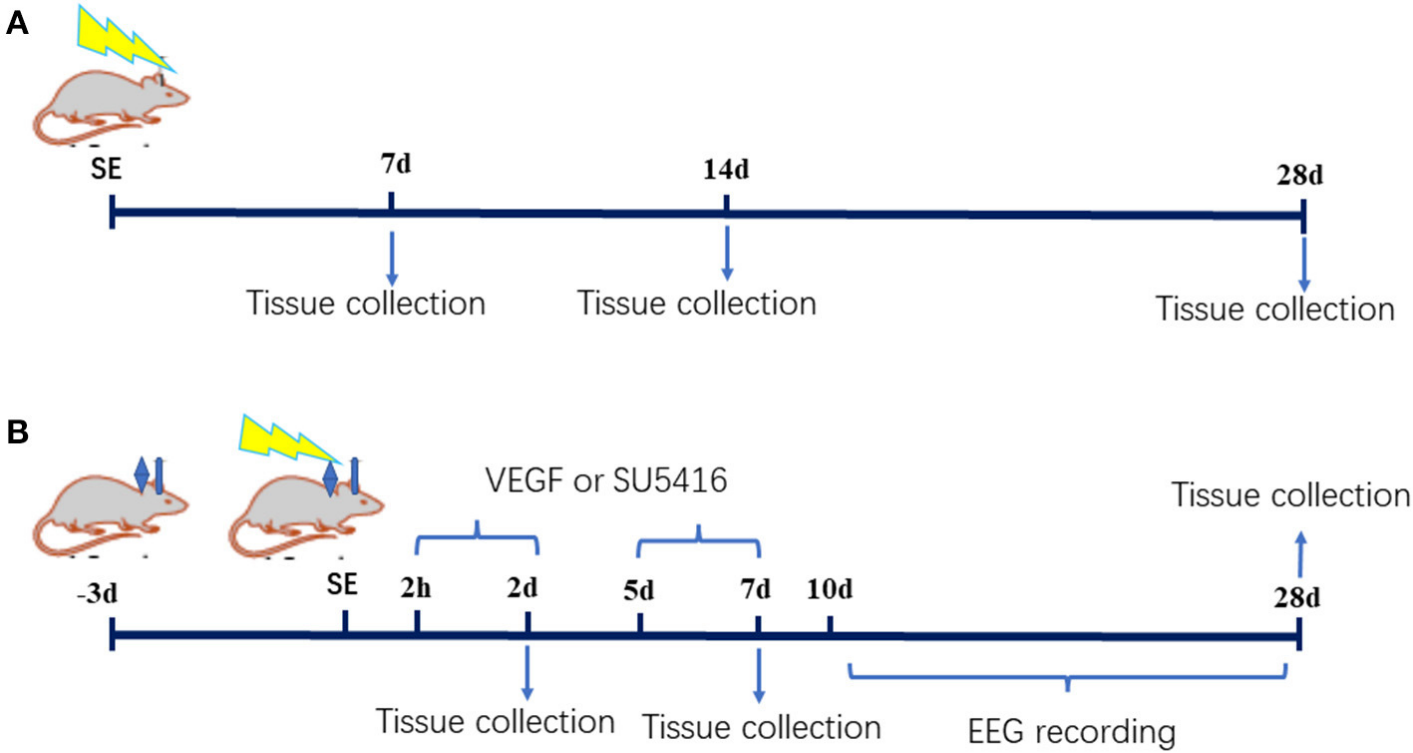
Animal experiment procedure. **(A)** The first part **(B)** the second part.

The study was divided into two parts. In the first part, thirty male postnatal day 20 rats were randomly divided into the SE (*n* = 15) and control groups (*n* = 15). Pilocarpine was administered in accordance with the protocol described above. The rats were sacrificed by decapitation under deep anesthesia with 10% chloral hydrate (3 ml/kg, i.p.) at various time points (7, 14 or 28 d after SE, *n* = 5 in each group at each time point). In the second part, ninety-five postnatal day 18 rats were randomly divided into the control group (*n* = 13), the SE group (*n* = 82), the acute-phase SE VEGF intervention (SV0) group (injection of VEGF into the lateral ventricle from 2 h to 2 d after SE), the acute-phase SE SU5416 intervention (SU0) group (injection of SU5416, a VEGFR2 inhibitor, into the lateral ventricle from 2 h to 2 d after SE), the latent-phase SE VEGF intervention (SV5) group (injection of VEGF into the lateral ventricle from 5 d to 7 d after SE), and the latent-phase SE SU5416 intervention (SU5) group (injection of SU5416 into the lateral ventricle from 5 d to 7 d after SE). A catheter was implanted into the lateral ventricle on the 18th day after birth, and SE was induced 21 d after birth. The model rats were administered VEGF or SU5416 via the catheter inserted into the lateral ventricle from 2 h or 5 d after SE induction. The rats were sacrificed 2, 7, or 28 d after SE (*n* ≥ 14 in each group). The experimental procedure is presented in Figure 1.

### Pump Implantation and Intracerebroventricular Drug Injection

The rats were anesthetized via ether inhalation and placed in a stereotactic frame. The anterior fontanelle was used as the origin. A Hamilton syringe was placed in the right lateral cerebral ventricle at predetermined coordinates: 1.2 mm posterior to and 1.2 mm lateral from lambda and a depth of 3.8 mm. A drill was used to create a small hole in the skull, a catheter was inserted (Roanoke Corporation, USA) into the lateral ventricle and fixed with glass ionomer cement, and a tube was inserted into the catheter to seal it after the cement solidified. After the operation was completed, the rats were placed in cages and kept warm. After the operation, an i.p. injection of 300,000 units/kg penicillin was administered to prevent infection.

Rats with SE were randomly divided into the SV0, SU0, SV5, and SU5 groups and received injection of VEGF 165 40 ng [8 ng/μl, dissolved in phosphate-buffered saline (PBS), Peprotech, Rocky Hill, USA] or SU5416 5 mM [1 mM/μl, dissolved in dimethylsulfoxide (DMSO), Selleck, USA] per rat for 3 continuous days (2 h, 1 d, and 2 d or 5 d, 6 d, and 7 d after SE) at a rate of 0.5 μl/min. After injection, the microsyringe was kept in place for 5 min until the liquid was fully diffused. Then, the injection needle was removed, and the cap was closed.

### Immunofluorescence and Immunohistochemistry

Anesthetized rats were perfused through the left ventricle with 4% paraformaldehyde. Hippocampal coronal slices were cut into consecutive sections at a thickness of 40 μm and immediately placed in cryoprotectant solution [150 g of C_12_H_22_O_11_ and 150 ml of (CH_2_OH)_2_ dissolved in 500 ml of PBS] at 4°C until immunofluorescence was performed. The following primary antibodies were used: anti-VEGF (rabbit polyclonal antibody, Abcam, 1:200), anti-VEGFR2 (rabbit polyclonal antibody, Abcam, 1:200), anti-Dcx (rabbit polyclonal antibody, Abcam, 1:200), anti-CD31 (mouse monoclonal antibody, Abcam, 1:200), and anti-polysialic acid-neural cell adhesion molecule (PSA-NCAM) (mouse monoclonal antibody, Millipore, 1:80). Nis-element BR3.2 software was used to capture images of the hippocampal DG area and blindly analyzed the average fluorescence intensity from 2 sections per animal (*n* = 5 for each group) selected randomly.

Brain tissues were embedded in paraffin after dehydration, and the paraffin sections were cut coronally at a thickness of 4 μm. The sections were soaked in xylene for at least 1 h for dewaxing and then blocked in 5% BSA. A CD31 primary antibody (mouse monoclonal antibody, Abcam, 1:200) was used, DAB was used for color development (Zhongshan, China), and the sections were counterstained with hematoxylin. The slices were imaged under a microscope with an oil immersion lens. Vessels with a diameter of <10 μm were considered microvessels. The microvessel density (MVD) in the hilar area of the DG was calculated with NIS software according to the following formula: MVD = number of microvessels (N)/area of interest (mm^2^). The averaged MVD from 4 sections per animal (*n* = 5 for each group) selected randomly was assessed in each group.

### Western Blot Analysis

The hippocampus of each rat was rapidly dissected and stored in liquid nitrogen immediately after dissection. Protein concentrations were determined using a Bio-Rad protein assay kit (Bio-Rad Laboratories, USA). An equal amount (30 μg) of each sample was loaded on 6% and 10% SDS polyacrylamide gels. The proteins were separated by electrophoresis and then transferred to polyvinylidene difluoride membranes (0.22 μm, Millipore Corp, Billerica, MA, USA). The membranes were blocked with PBS containing 10% milk for 1 h and then incubated with the following specific primary antibodies overnight at 4°C: anti-VEGFR2 (rabbit polyclonal antibody, Abcam, 1:1000), anti-AKT (rabbit monoclonal antibody, Abcam, 1:2000), anti-phospho-akt (rabbit monoclonal antibody, Cell Signaling Technology, 1:2000), anti-ERK (rabbit monoclonal antibody, Cell Signaling Technology, 1:2000), and anti-phospho-erk (rabbit monoclonal antibody, Cell Signaling Technology, 1:2000). In parallel, the Western blots were probed with an anti-β-actin antibody (mouse monoclonal antibody, 1:500, Boster) for data normalization. The proteins were visualized using Clarity Western Electrochemiluminescence (ECL) Substrate (Bio-Rad, USA), and the gray values were analyzed using a Syngene imaging system.

### Nissl Staining

Samples were processed in the same way as described for immunohistochemistry, and paraffin sections were cut coronally at a thickness of 5 μm and subjected to Nissl staining. The sections were stained with cresyl violet for the subjective evaluation of hippocampal damage.

### Electroencephalography

A 1.00-mm-diameter hole was made in the parietal skull of each rat (*n* = 3 for every group) on the left and right sides of the sagittal suture, and a hole was drilled above the telencephalon for grounding. Then, simple homemade electrodes with 0.1-mm nickel-chromium wires were inserted so that the wires touched the dura mater for scalp EEG (Supplementary Figure 1). After 3 days of recovery, SE was induced in rats to establish the SE model, and EEG was performed during modeling and then for 2 h per day until 28 days after SE with a radiophysiological signal detection system (Telemetry ReSCarch Limited, New Zealand). The rats in the other intervention groups were subjected to EEG for 2 h per day for 18 days starting after drug administration (10 days after SE). EEG was mainly used to record changes in SRS. An ADL instrument amplifier was used with the following parameters: low pass filter at 100 Hz, digitization rate of 2 kHz and amplification rate of 1,000 times. According to a previous study (24), seizures were defined as discharges with a frequency >5 Hz, an amplitude >2 times than baseline and a duration of 10 s. pClampfit software was used to record the average number of daily spontaneous seizures, identify the 10-min period with the most severe seizures, and analyze the frequency of seizures and the duration of each seizure in a 10-min period.

### Statistical Analysis

All data are presented as the means ± SD and were analyzed with SPSS 17.0 statistical software. The data were analyzed using independent sample Student's *t*-tests or one-way analysis of variance (ANOVA) followed by the LSD *post hoc* test or Dunnett's T3 test. *p* < 0.05 was considered statistically significant.

## Results

### SE Induction and EEG Recording

According to Racine's five category classification of seizures (29), SE was successfully induced in a total of 111 rats after pilocarpine administration, and the SE was interrupted after 60 min by injection with diazepam. Fourteen rats that failed to develop SE or died during SE were excluded. EEG recording was performed to detect epileptic discharge from SE to epileptogenesis (**Figure 7A**). The electroencephalograms of SD rats in the control group mainly consisted of α and β waves, with scattered θ waves and no SRS. Lithium chloride-pilocarpine induced SE in immature rats, and the electroencephalograms of these rats mainly showed persistent high-amplitude sharp waves and spike waves during convulsive states. However, in the latent phase after SE, electroencephalograms were almost normal, showing a few sharp waves and spikes. In the chronic phase, electroencephalograms mainly showed scattered sharp waves and spine waves lasting from a few seconds to tens of seconds, which represented spontaneous repeated epileptic discharges. The rats exhibited stage 2–4 epileptic seizures on the Racine scale, as unilateral or bilateral forelimb clonus was common during the seizures, and generalized convulsions were rare.

### Relationship Between NSCs and Neovascularization

**Figure 2:**
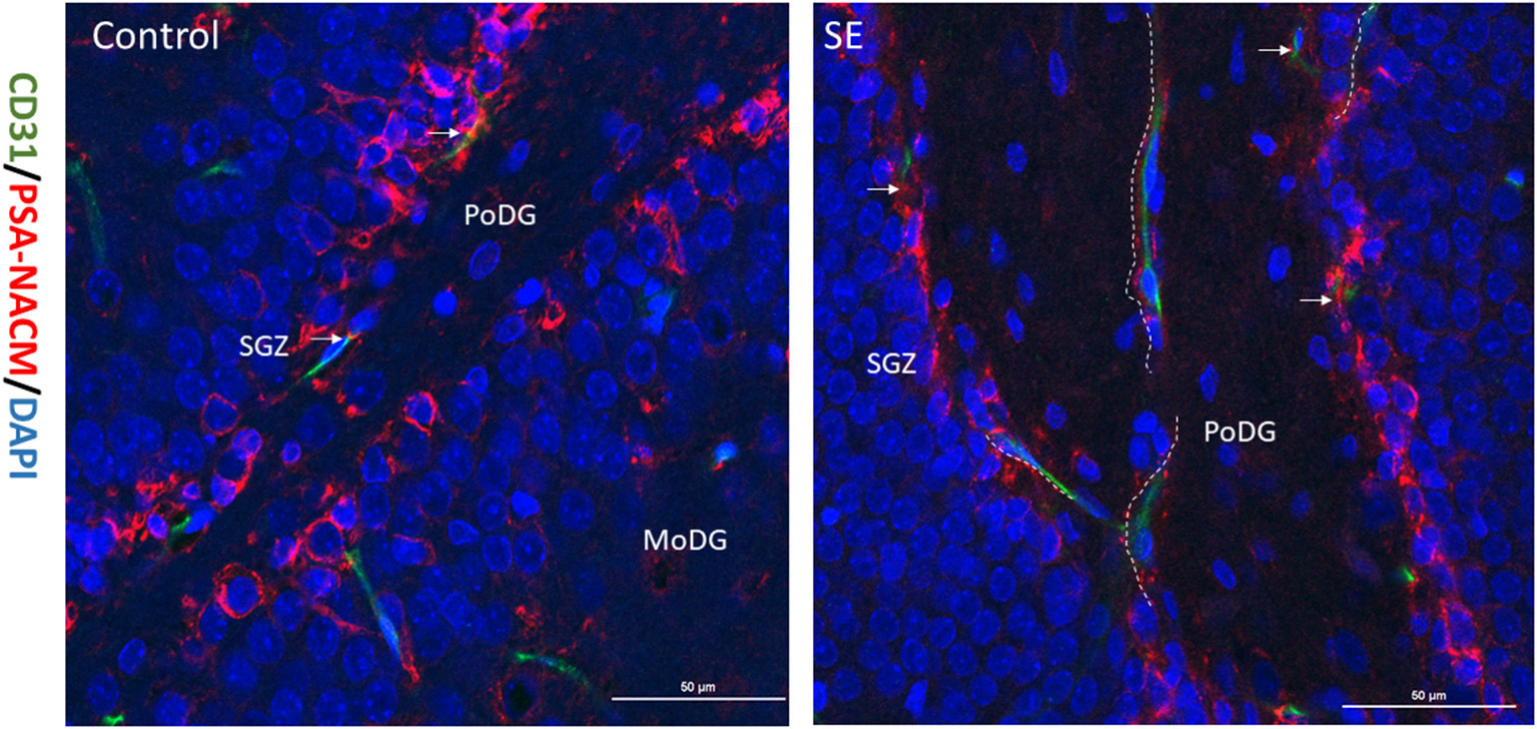
Relationship between migrated NSCs and neovascularization. The expression of CD31 and PSA-NACM in the hippocampal DG at 7 days post SE in immature rats. PSA-NACM was stained prominently in the granule cells of SGZ (arrows) and is tributions of CD31 at the polymorphic layer of the DG (PoDG) or molecular layer of the DG (MoDG) in the hippocampus. PSA-NACM colocalized with CD31 expressed in hundreds of microns in the direction of PoDG after SE. It indicated that NSCs migrated hundreds of microns from the SGZ in the direction of new blood vessels. CD31, green; PSA-NACM, red; scale bar, 50 μm.

PSA-NCAM is a single-chain transmembrane glycoprotein that mediates cell-cell and cell-matrix adhesion and is a marker of NSCs migration. Double labeling immunofluorescence for PSA-NACM (red fluorescence) and CD31 (green fluorescence) was used to assess the spatial relationship between hippocampal NSCs and blood vessels in the latent period after SE. As shown in the merged image in Figure 2, CD31 colocalized with PSA-NACM are concentrated in the SGZ and arranged in chains along CD31 after SE. It indicates that NSCs along with newly blood vessel gradually migrating into the granule cell layer (GCL) even arrived at molecular layer of the DG (MoDG) or the polymorphic layer of the DG (PoDG) from SGZ after SE.

### Effect of Differential Regulation of VEGF During Different Time Periods on Neurogenesis During Epileptogenesis After SE

**Figure 3:**
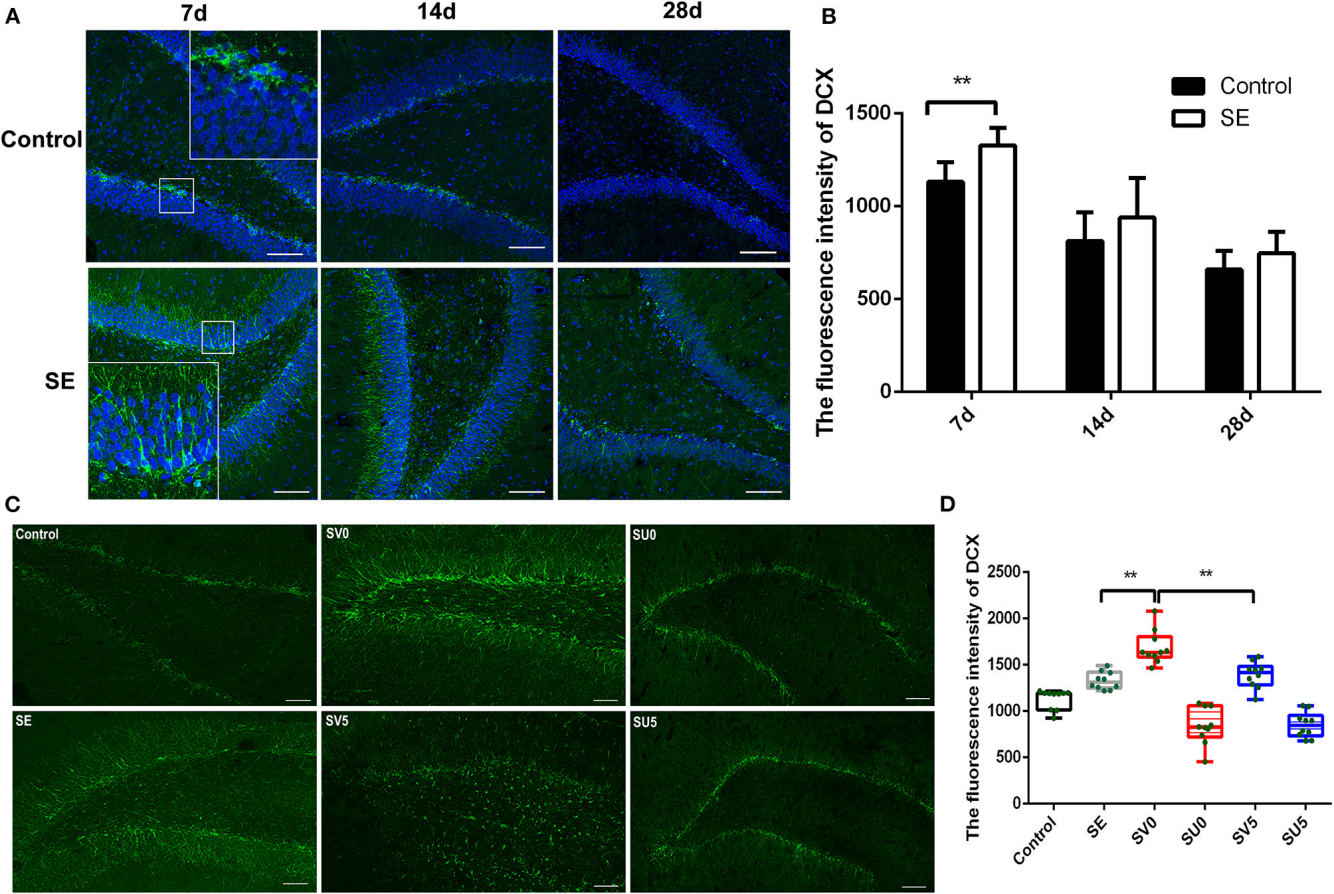
The effect of VEGF on neurogenesis during epileptogenesis after SE **(A)** The immunofluorescence of DCX in the DG of the hippocampus after SE (Dcx: green; DAPI: blue; 200 ×, scale bar: 100 μm). **(B)** Dcx fluorescence intensity in the DG of the hippocampus after SE. The mean fluorescence intensity of DCX prominently increased at 7 days post SE compared to the control, **P < 0.01. **(C)** Effect of VEGF-based interventions on neurogenesis in different phases after SE (SV, VEGF injection; SU, application of a VEGFR2 inhibitor. Dcx: green, scale bar: 100 μm). **(D)** Dcx fluorescence intensity in the VEGF intervention groups in different phases after SE. Values were mean ± SD, **P < 0.01, *n* = 5 for each group.

Doublecortin (Dcx) is a specific marker of neuronal precursor cells that can act on tubulin to polymerize microtubules, stabilize the cytoskeleton, and directly impact the initiation of neuronal migration. Immunofluorescence was conducted to assess the dynamic changes in Dcx expression from the latent period to the chronic period of epileptogenesis after SE (Figure 3A). Dcx (green fluorescence) was expressed on the cell membrane in the shape of a flocculent ribbon or ring and was mainly expressed in the hippocampal SGZ in a chain arrangement. In the control group, almost all of the Dcx-positive cells in the hippocampus were distributed in the hippocampal SGZ. The fluorescence intensity of Dcx in the hippocampal DG area showed a decreasing trend from 28 to 49 days after birth, which was consistent with the process of brain development. Dcx expression also gradually decreased from 7 to 28 days after SE in the SE group, but the Dcx fluorescence intensity was higher in the SE group than in the control group at each time point, and the Dcx fluorescence intensity was significantly higher in the SE group than in the control group at 7 days after SE. The difference was statistically significant (control: 1,132.03 ± 106.10; SE: 1,328.69 ± 94.81, *P* < 0.01), as shown in Figure 3B. In addition, Dcx-positive cells were not confined to the SGZ in the SE group. Dcx-positive cells were distributed in the GCL of the DG at 7 days after SE and showed a tendency to migrate across the GCL to the PoDG. This finding suggests that NSCs that proliferate in the acute phase after SE tend to migrate during the latent period and that the latent period is critical for neurogenesis and the migration of NSCs (Figure 3A).

We further interfered with the expression of VEGF in the acute and latent phases after SE and evaluated the migration of NSCs (Figure 3C). ANOVA revealed a significant effect of VEGF at different time periods on DCX [F_(5, 54)_ = 50.746, *P* < 0.001]. In the SV0 group, Dcx was still mainly located in the SGZ and GCL, but the fluorescence intensity of Dcx was significantly higher in the SV0 group than in the SE group, and the difference was statistically significant (*P* < 0.001). In the SV5 group, Dcx-positive cells were not confined to the SGZ and GCL, and many Dcx-positive cells were scattered in the PoDG and MoDG. The Dcx fluorescence intensity was significantly lower in the SV5 group than in the SV0 group (*P* < 0.01), but the difference in Dcx fluorescence intensity between the SV5 group and the SE group was not significant. However, in the SU0 and SU5 groups, Dcx-positive cells were mainly distributed in the SGZ, and the fluorescence intensity was much lower in the SU0 and SU5 groups than in the SE group (Figures 3C,D). These data suggest that promotion of VEGF expression in the acute phase after SE can significantly promote the proliferation of NSCs, that the promotion of VEGF expression during the latent phase after SE may lead to abnormal migration of NSCs. Becides, the inhibition of VEGF expression in the acute phase or the latent phase after SE may inhibit neurogenesis.

### Effect of Differential Regulation of VEGF During Different Time Periods on Microvascular Regeneration During Epileptogenesis After SE

**Figure 4:**
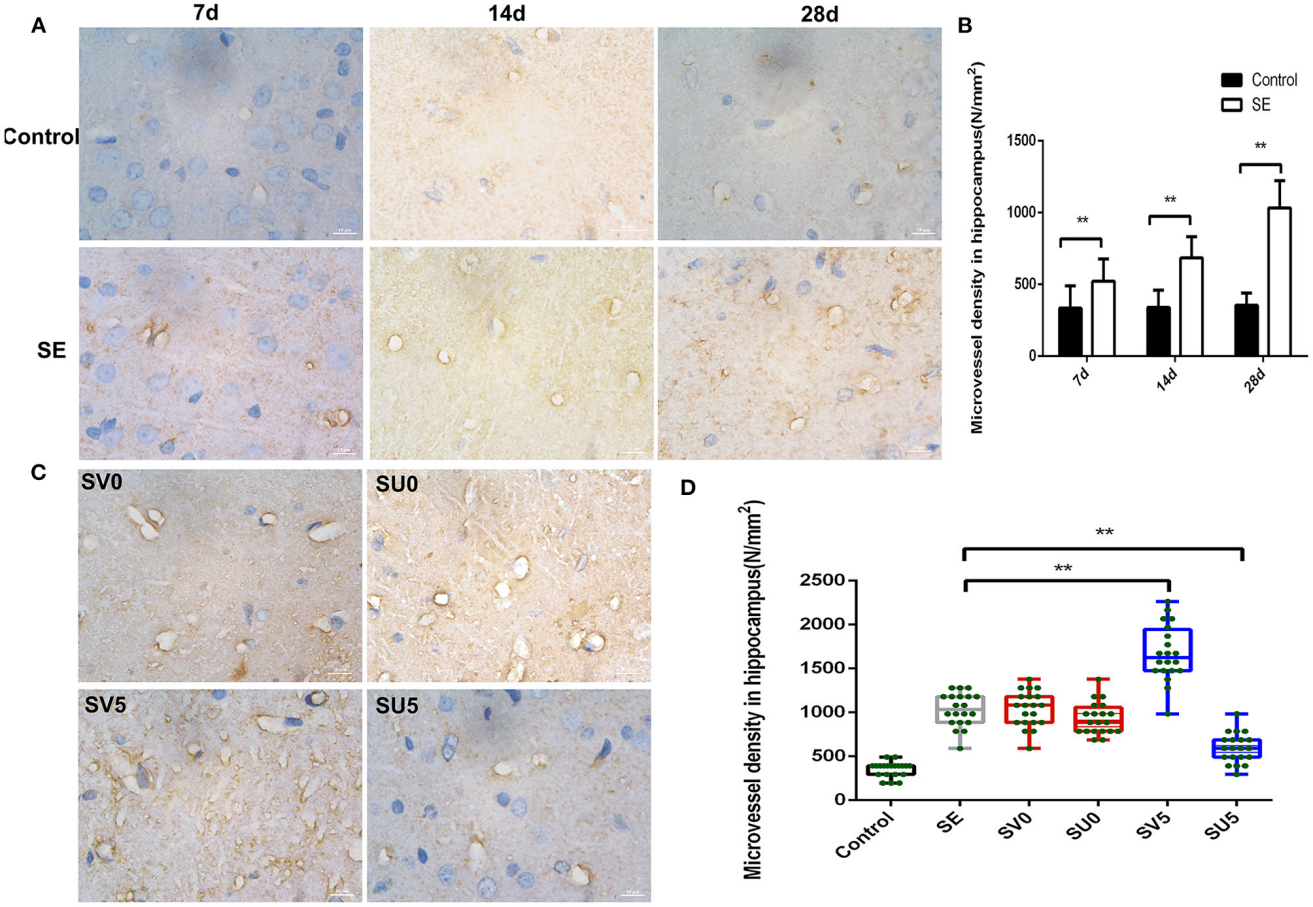
Effect of VEGF on microvascular regeneration during epileptogenesis after SE. **(A)** Expression of CD31 in the hippocampus after SE (1,000 ×, scale bar: 10 μm). **(B)** MVD in the hippocampus after SE (N/mm2). The MVD in the SE group was significantly higher at each time point than that in the control group, **P < 0.01. **(C)** Effect of VEGF intervention in different phases after SE on microvascular regeneration (1,000 ×, scale bar: 10 μm). **(D)** MVD in the hippocampus after VEGF intervention in different phases after SE. The MVD of SV5 was much higher than that of the SE group; however, the MVD of SU5 was much lower than that of the SE group. **P < 0.01, *n* = 5 for each group. SV, VEGF injection; SU, application of a VEGFR2 inhibitor.

Platelet endothelial cell adhesion molecule-1 (PECAM-1/CD31), which is present in the tight junctions of vascular endothelial cells, is a marker of neovascularization. Immunohistochemistry was used to detect the dynamic changes in CD31 expression from the latent phase to the chronic phase after SE. The results, which are shown in Figure 4A, showed changes in microvascular regeneration.

During the development of the immature brain (28 d-49 d after birth), the expression of CD31 in the hippocampus was not significantly correlated with age in the control group, while the expression of CD31 in the hippocampal DG gradually increased from 7 to 28 days after SE in the SE group. The microvessel density (MVD) in the hilar area of the DG was calculated with NIS software according to the following formula: MVD = number of microvessels (N)/area of interest (mm2). The MVD in the SE group was significantly higher than that in the control group at each time point, and the difference was statistically significant (Control-7d: 334.62 ± 154.46, SE-7d: 521.62 ± 156.75, *P* < 0.01; Control-14d: 339.54 ± 121.48, SE-14d: 684.01 ± 147.97, *P* < 0.01; Control-28d: 354.30 ± 86.86, SE-28d: 1033.39 ± 190.25, *P* < 0.01), as shown in Figure 4B. This result suggests that vascular proliferation occurs during the latent phase after SE, gradually increases during epileptogenesis, and peaks in the chronic phase after SE.

We further interfered with the expression of VEGF in the acute and latent phases after SE and investigated the changes in the density of new microvessels in the hippocampus in the chronic phase after SE (Figures 4C,D). ANOVA revealed a significant effect of VEGF at different time periods on CD31 and MVD [F_(3, 76)_ = 79.087, *P* < 0.001]. However, the expression of CD31 and MVD in the hippocampus in the chronic phase after SE was not significantly different between the groups treated in acute-phase SE (the SV0 and SU0 groups) and the SE group. The expression of CD31 in the chronic phase after SE and the MVD were significantly higher in the SV5 group, in which intervention occurred in the latent phase after SE, than in the SE group. The difference was statistically significant (*P* < 0.01). This finding suggests that the best time window for regulating microvessel regeneration after SE is the latent phase. Inhibition of VEGF expression in the latent phase after SE can inhibit the regeneration of microvessels after SE.

### Molecular Mechanism of VEGF Regulation in Different Phases After SE

**Figure 5:**
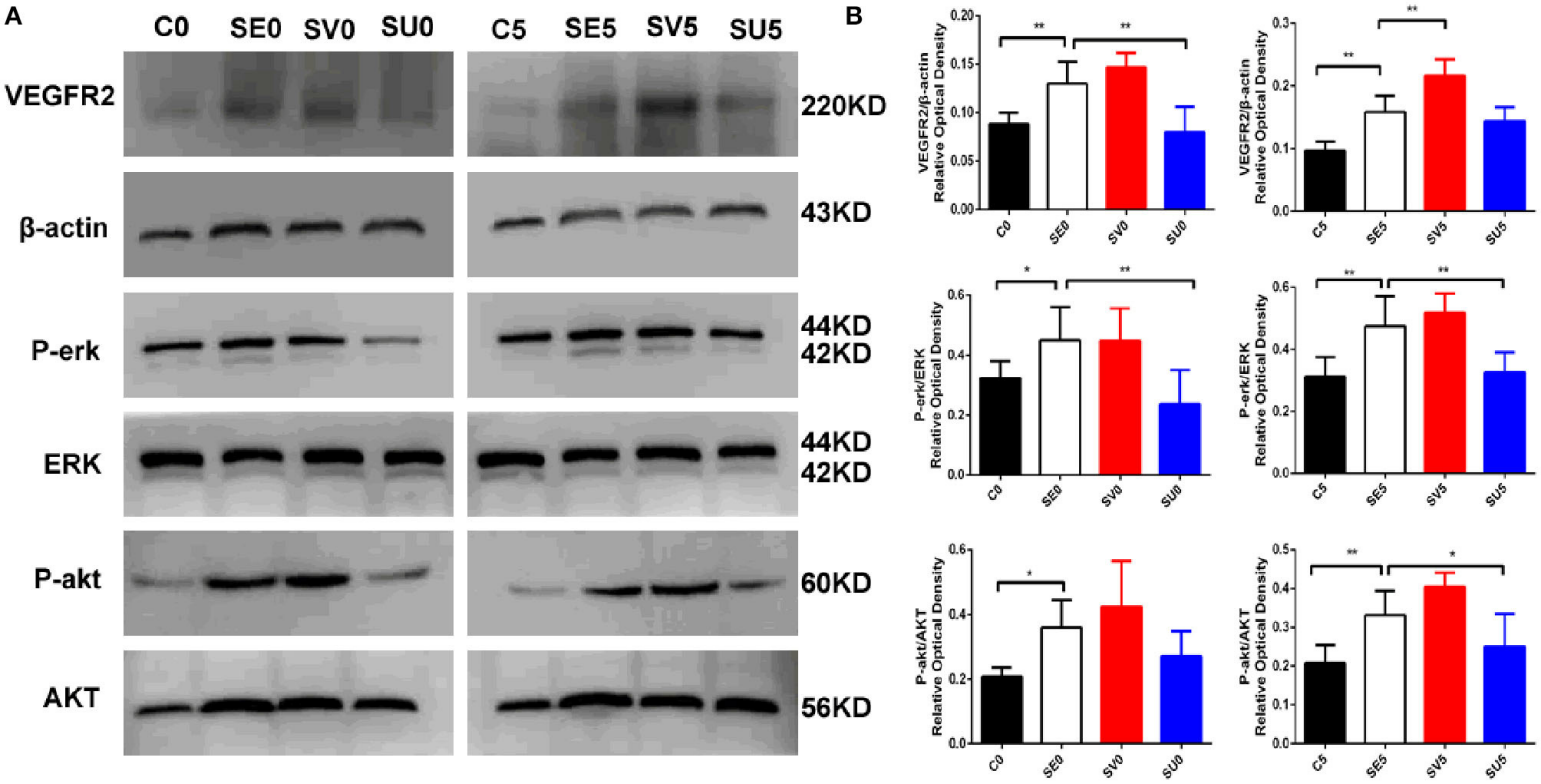
Western blotting results showing the effects of VEGF regulation in different phases after SE on downstream signaling pathways. **(A)** Representative results with the corresponding antibodies. VEGFR2, P-akt, P-erk, AKT and ERK expression levels in the hippocampi of SE rats following regulation of VEGF in different phases after SE. **(B)** Quantitative analyses of the protein expression level in each group. The relative optical densities of VEGFR2, P-erk and P-akt were upregulated post-SE *P < 0.05, **P < 0.01, *n* = 6 for each group. C, control; SV, VEGF injection; SU, application of a VEGFR2 inhibitor.

Western blotting was used to determine the activation status of VEGFR2, phospho-protein kinase B (P-akt), and phospho-extracellular signal-regulated kinase (P-erk) to study the influence of VEGF regulation in different stages after SE on its downstream signaling pathways. The expression levels of kinase B (AKT) and extracellular signal-regulated kinase (ERK), which are downstream of the VEGFR2 protein, were significantly higher in the SE group than in the control group. The protein expression of VEGFR2 in the hippocampus was upregulated in the SV0 group and SV5 group compared with the SE group [F_(3, 20)_ = 29.801, *P* < 0.001], and the expression levels of the downstream proteins P-akt [F_(3, 20)_ = 6.270, *P* = 0.004] and P-erk were increased to varying degrees [F_(3, 20)_ = 6.581, *P* = 0.003]. VEGFR2 and P-erk protein levels in the SU0 group were significantly lower than those in the SE group, and the difference was statistically significant (*P* < 0.01). P-erk and P-akt protein levels in the SU5 group were significantly lower than those in the SE group, and the difference was statistically significant (*P* < 0.01, *P* < 0.05), as shown in Figure 5. The results indicate that the AKT and ERK signaling pathways are activated in the acute phase after SE. The AKT and ERK signaling pathways downstream of VEGFR2 are activated to varying degrees and may participate in epileptogenesis.

### Nissl Staining for the Assessment of Hippocampal Structure in the Chronic Phase After SE

**Figure 6:**
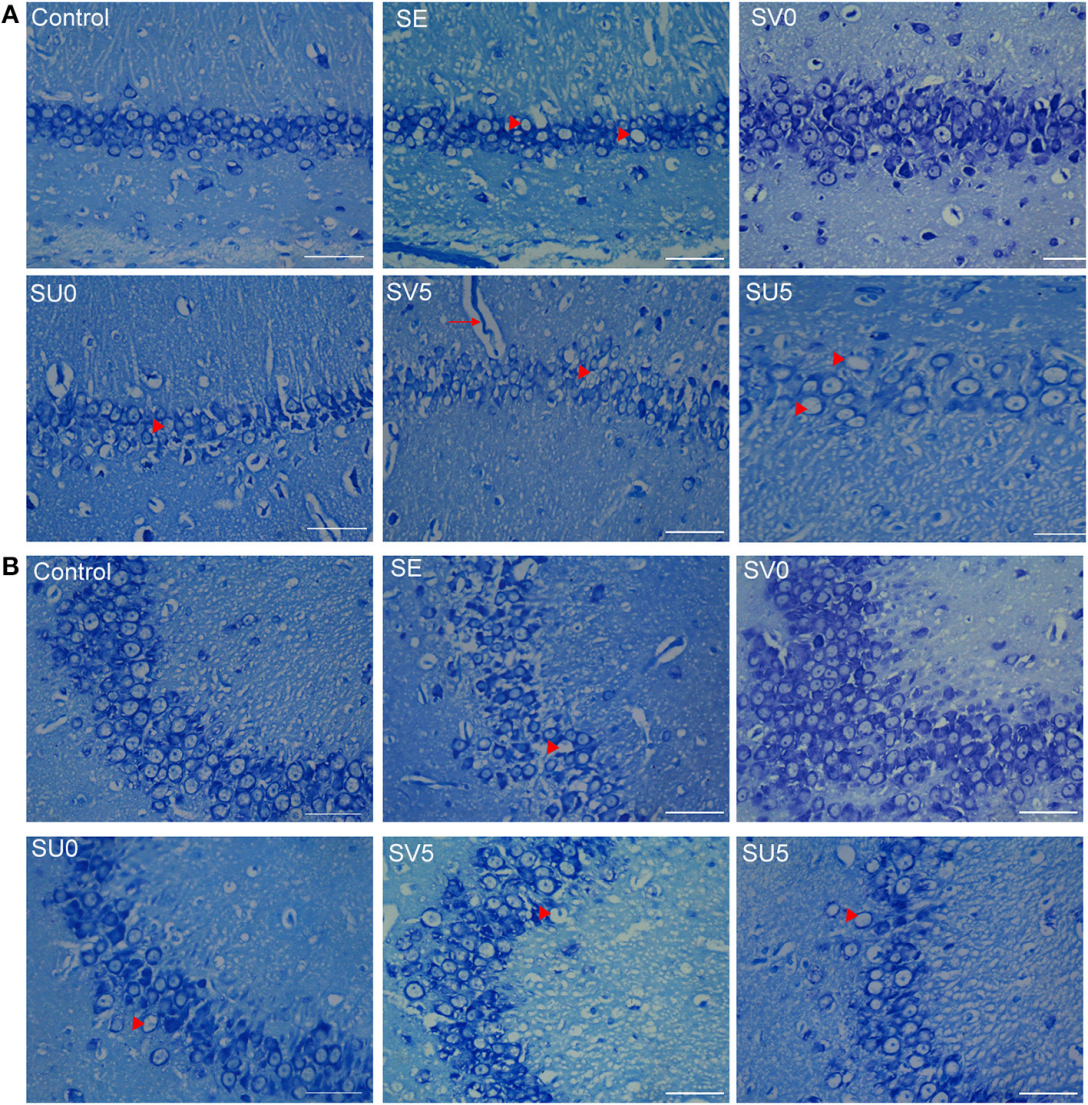
Morphological structure of the hippocampus in different groups in the chronic phase after SE. **(A)** Morphological structure of the hippocampal CA1 region and **(B)** CA3 region in the different groups in the chronic phase after SE. Vascular infiltration was observed in the CA1 region in the SV5 group, as shown by arrows (scale bar: 50 μm).

As shown in Figure 6, in the control group, the size and morphology of neurons in the CA1 and CA3 regions of the hippocampus were neurons; the neurons were neatly and closely arranged and structurally intact, the Nissl bodies were clearly visible, and there was no obvious neuronal loss. The hippocampal structure was disrupted in the chronic phase after SE, the cell arrangement was loose, the CA1 and CA3 regions were missing, Nissl bodies were missing, and scattered nuclear lysis was observed (shown as arrowhead). In the SV0 and SV5 groups, the pyramidal cell of CA1 and CA3 areas were almost tightly organized and ordered. The Nissl bodies were visible in the SV0 group. In addition, vascular infiltration was observed in the CA1 region in the SV5 group (shown as arrow). However, in the SU0 and SU5 groups, the CA1 and CA3 regions showed obvious Nissl bodies and neuronal loss that were same as observed SE group.

### EEG Manifestations in Different Phases of Epileptogenesis in Immature Rats

**Figure 7:**
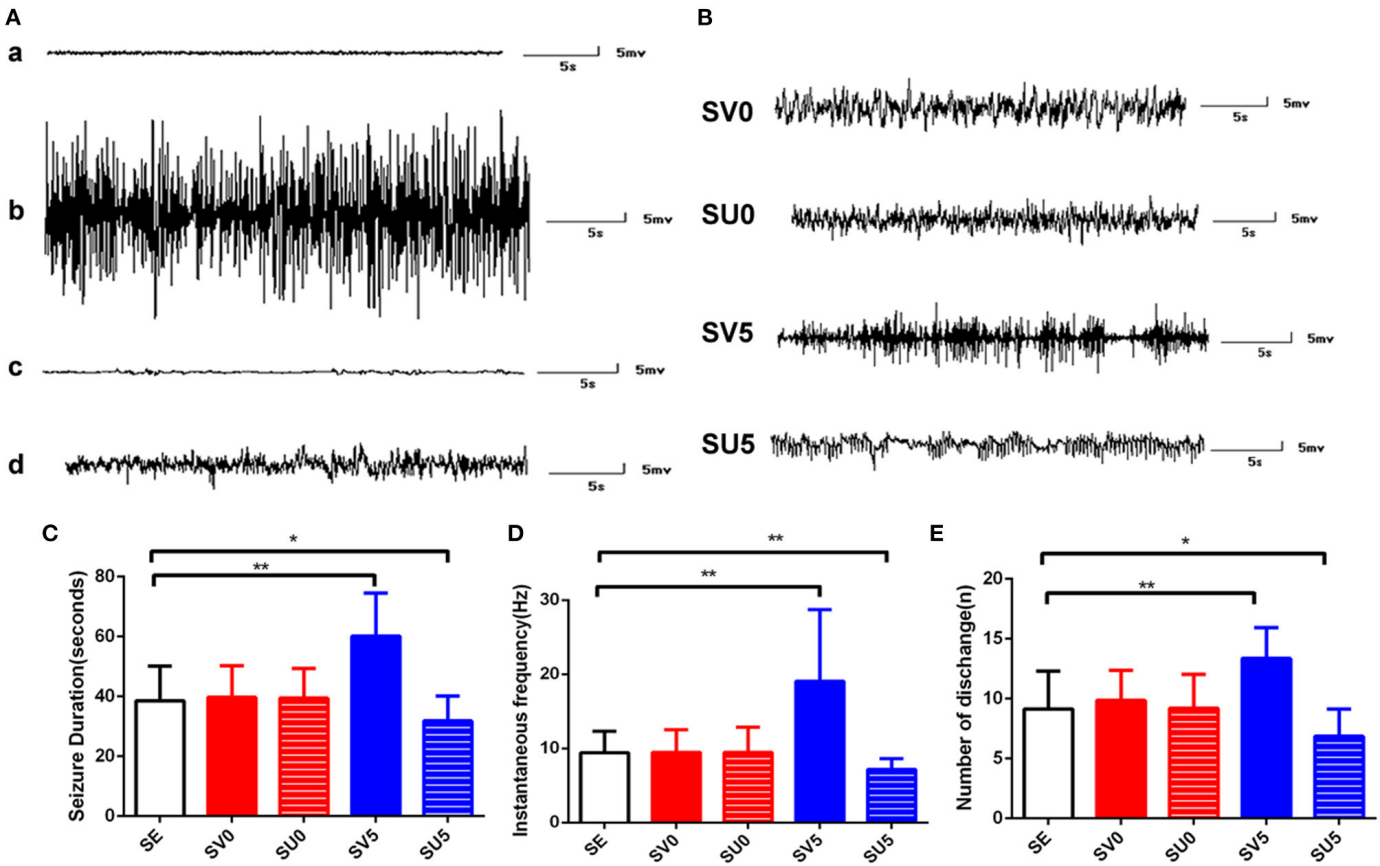
EEG recordings of rats from different groups during different periods. **(A)** EEG recordings from rats in the SE group during epileptogenesis. (a) EEG recording from a normal immature rat. (b) EEG recording from a rat in the acute phase after SE. (c) EEG recording from a rat in the latent period of epileptogenesis after SE. (d) EEG recording of SRS in the SE group. **(B)** EEG recordings of SRS in the different intervention groups. **(C)** SRS duration, **(D)** seizure frequency and **(E)** the number of discharges during the SRS phase after SC. *P < 0.05, **P < 0.01.

EEG discharges were recorded daily in the different intervention groups during epileptogenesis from 10 to 28 days after SE, and spontaneous epileptic discharges of varying degrees, which manifested as scattered sharp waves and spike waves lasting tens of seconds, were detected in all experimental rats in the chronic phase after SE (Figure 7B). This result indicates that inhibiting or promoting VEGF expression in different phases after SE does not significantly reduce or increase the incidence of epileptic discharges in the chronic phase after SE.

The duration, frequency and average number of SRS per day in each intervention group were further analyzed. The treatment factor had a significant effect on the seizure duration [F_(4, 190)_ = 35.918, *P* < 0.0,01], seizure frequency [F_(4, 160)_ = 28.851, *P* < 0.001], and number of discharges [*F*_(4, 65)_ = 10.737, *P* < 0.001]. There were no significant differences between the SV0 and SU0 groups and the SE group; however, the duration, frequency, and average number of seizures per day were significantly higher in the SV5 group than in the SE group, and the difference was significant (*P* < 0.01). The main behavioral manifestations of SV5 group rats during SRS were facial twitching, rhythmic nodding and chewing, general rigidity, raised unilateral forelimb clonus, and bilateral forelimb clonus with hindlimb standing in the most severe cases. Interestingly, the average duration of each seizure (*P* < 0.05), seizure frequency (*P* < 0.01), and average number of seizures per day (*P* < 0.05) were significantly lower in the SU5 group than in the SE group, and the difference was statistically significant. Based on observation by the naked eye, the behavioral manifestations, which mainly included spontaneous eye gaze and facial muscle twitching and rarely included unilateral or bilateral forelimb clonus, were significantly less severe in the SU5 group than in the SE group. These results suggest that regulation of VEGF expression in the latent phase after SE has a greater impact on the degree of epilepsy. Inhibiting the expression of VEGF in the latent phase after SE can reduce the severity of EEG epileptic discharges.

## Discussion

SE is more likely to impair the development of neural networks and thus lead to intractable epilepsy in infants and young children than in older individuals. In general, pathological changes in the brain occur in acute, latent phase and chronic phases after convulsive brain injury (3). Neurogenesis and remodeling of microvessels are important role in the process of epileptogenesis (17).

Studies have found that hippocampal neurogenesis can be induced within at least 1–2 weeks after an acute seizure. In turn, the SRS could also stimulate the proliferation of NSCs in the DG (30–33). New neurons in the subventricular zone (SVZ) of the adult hippocampus migrate only a short distance to the GCL to replace apoptotic neurons and reintegrate with neurons in the GCL under normal conditions. However, in the mature brain after SE, the NSCs show abnormal migration, with neurons directly entering the DG or even passing through the GCL to enter the MoDG. The axons of mature neurons in the MoDG follow the pathway of moss fibers of granular cells in the hippocampal DG into the CA3 region after SE, forming abnormal synaptic connections with CA3 neurons (34). The synapses formed by moss fibers exhibit high excitability and low inhibition and are prone to abnormal synchronous discharges; thus, they increase excitability, lead to SRS, form abnormal excitatory neural circuits associated with epilepsy and are the pathological basis of refractory temporal lobe epilepsy (35, 36).

Our data suggest that the latent phase of SE is a critical period for the migration of NSCs in the immature brain. Unfortunately, early migration-related regulatory signals are often lacking or incorrect in this period and can thus easily cause ectopic migration of neurons instead of contributing to their correct integration in the GCL as in the mature brain. Besides, the ectopic NSCs destined for apoptosis even before integrating into the DG circuit during the acute period post-SE. Besides, it has a widely recognized that vascular regeneration is involved in epilepsy pathogenesis (14, 37). In our study, we also found that seizures could affect microvessel proliferation that the density of hippocampal microvessels gradually increased after the incubation period and continued to increase until the chronic phase. According to our data, neovascularization acted as a stent for NSC migration, and pathological angiogenesis affected the direction of migration and abnormal integration of NSCs and contributed to the development of epilepsy, which is consistent with previous research (13, 17, 38). If the survival of hippocampal NSCs is protected during the acute period after SE and the regeneration of microvessels is inhibited during the latent period after SE, the formation of epileptic networks could be prevented.

The interaction of VEGF has been indicated to be involved in neurogenesis and angiogenesis in normal adult brain. It has been confirmed in cerebral ischemia models that VEGF has different effects in different periods after brain injury. Blocking VEGF expression in the early stage of ischemia can reduce cerebral edema. Promoting VEGF expression in the subacute stage can improve cerebral vascular perfusion. VEGF can improve cerebral vascular perfusion 48 h after cerebral ischemia and can promote angiogenesis and coordinate neurogenesis at the injury site but does not further increase or disrupt vascular penetration (39). A study (40) revealed that injection of exogenous VEGF into the lateral ventricle in the subacute stage after brain injury, i.e., 7 days after successful modeling of stroke in neonates, promotes the proliferation of endothelial cells, increases the total number of blood vessels and protects the brain. Our previous studies showed that the peak expression of VEGF in the hippocampus was in the acute phase after SE, which promotes the proliferation of NSCs and reverses cognitive deficits (8). Considering the dual role and time-dependent effects of VEGF in pathological changes after brain injury, safe and practical application of VEGF after SE is the key to treatment. The results of our study could identify the appropriate time window for regulating vascular regeneration by targeting VEGF after SE.

In this study, by regulating the expression of VEGF in different periods after SE, we found that upregulation of VEGF expression in the acute phase after SE significantly promotes the proliferation of NSCs and can significantly prevent the loss of neurons in the CA1 and CA3 regions but has no obvious effect on microvascular regeneration. However, promoting the expression of VEGF during the latent period after SE further aggravates the ectopic migration of NSCs and significantly increases MVD. However, if VEGFR2 is blocked in the acute phase, it has little effect on the proliferation of NSCs and no obvious effect on the microvasculature. Blocking VEGFR2 in the latent phase can inhibit the remodeling of blood vessels and the ectopic migration of NSCs.

SRS emergence is a key indicator of the occurrence and development of epilepsy, and our data showed that downregulation of VEGF expression in the latent phase after SE could effectively reduce the severity of epileptic discharges during SRS by shortening the duration of each seizure and reducing the frequency and number of seizures, suggesting that the key regulatory period of epileptogenesis is the latent phase. This finding is consistent with previous reports (28, 41) that selective inhibition of neurogenesis after SE would be valuable to reduce the severity of SRS. The present study also indicated that timig is very important to regulating the frequency and severity of SRS. We speculated that in the latent phase after SE, VEGF expression is inhibited, the level of hippocampal newly ectopic cell could be reduced, and these changes may interfere with the formation of abnormal excitatory synaptic connections and epileptic networks.

VEGFR2 is a tyrosine kinase receptor that can activate multiple downstream signaling pathways, causing neurogenesis, angiogenesis, neural circuit reconstruction and abnormal neural network formation. The PI3K/Akt signaling pathway and the MAPK/ERK signaling pathway are important signaling pathways in cells that play an important role in cell proliferation, differentiation, and apoptosis (42, 43). Both of these pathways may play a role in the development of epilepsy. A previous study (44) showed that the key pathophysiological mechanism underlying the ability of VEGF to protect against focal cerebral ischemia and increase the permeability of the BBB is the PI3K/Akt signaling pathway. Researchers (45) found that electrical convulsive stimulation can induce VEGF expression to regulate P-akt in the treatment of acute cerebral infarction. In addition, a previous study revealed that ERK is activated immediately after severe convulsions and that P-erk expression is upregulated and maintained at a high level from 28 d to 3 months after SE (46). In fact, it has been found that the crosstalk exists between AKT and ERK pathways. In the hippocampus, AKT could be activated first and then initiate ERK pathway to induct cell proliferation (47–49). In this study, the AKT and ERK signaling pathways were activated to varying degrees when VEGF/VEGFR2 signal transduction was interfered with in the acute phase or the latent phase after SE. This finding indicates that the molecular mechanism by which VEGF regulates hippocampal neurogenesis and vascular regeneration after SE may be these two signaling pathways, which are downstream of VEGFR2.

In summary, the present study demonstrated that VEGF, as a common target in nerves and blood vessels, might be closely related to neurogenesis and vascular regeneration in the immature brain during the development of epilepsy after SE. VEGF and VEGFR2 receptor may be valuable therapeutic targets for the prevention of epilepsy. An important aspect may be the proper therapeutic timing. Selective promotion the proliferation of hippocampal NSCs and inhibition of neurogenesis after SE, alleviation of the loss of neurons in the hippocampal and ectopic migration of newborn nerve cells caused by pathological regeneration, which contributes to effectively alleviate SRS severity. The upstream and regulatory factors of VEGF and the mechanism by which VEGF-mediated neovascularization affects the migration of NSCs were not investigated in this study and need to be further explored.

## Data Availability Statement

The original contributions presented in the study are included in the article/Supplementary Material, further inquiries can be directed to the corresponding author.

## Ethics Statement

The animal study was reviewed and approved by the Animal Care Committee of Chongqing Medical University, Chongqing, China.

## Author Contributions

WH and LJ conceived and designed the experiments. WH and HC performed the experiments and analyzed the data. XS, TL, and LC contributed reagents, materials, and analytical tools. WH wrote the manuscript. All authors contributed to the article and approved the submitted version.

## Funding

This research was funded by the National Natural Science Foundation of China, grant number NSFC Grant 81873792, the Science and Technology Research Program of Chongqing Municipal Education Commission, grant number Grant No. KJQN202100423, the Youth Basic Research Project from Ministry of Education Key Laboratory of Child Development and Disorders, grant number No.YBRP-202110, and Special Funding of Chongqing Postdoctoral Research Projects for WH.

## Conflict of Interest

The authors declare that the research was conducted in the absence of any commercial or financial relationships that could be construed as a potential conflict of interest.

## Publisher's Note

All claims expressed in this article are solely those of the authors and do not necessarily represent those of their affiliated organizations, or those of the publisher, the editors and the reviewers. Any product that may be evaluated in this article, or claim that may be made by its manufacturer, is not guaranteed or endorsed by the publisher.
