# Supplementary material for: VEGF Modulates Neurogenesis and Microvascular Remodeling in Epileptogenesis After Status Epilepticus in Immature Rats
Source: Front Neurol. 2021 Dec 24;12:808568. doi: 10.3389/fneur.2021.808568 (PMC8739962; doi:10.3389/fneur.2021.808568)
Supplement: Supplementary file 1 [file Presentation_1.pdf]

Supplementary Material

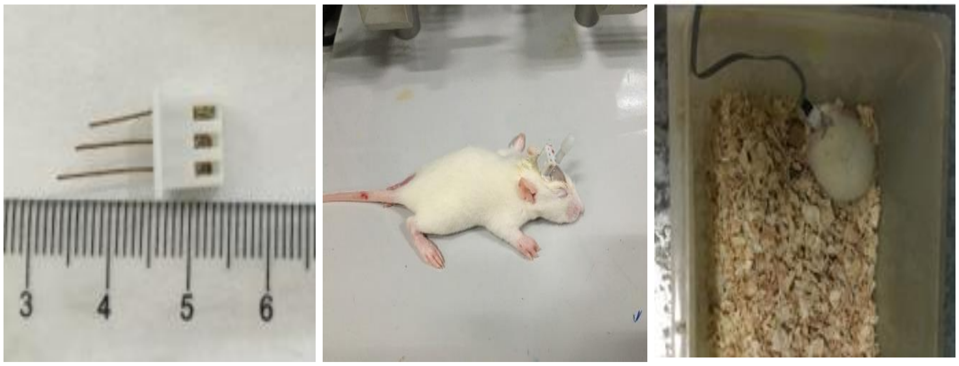

Supplementary Figure 1. Simple electrode installation and EEG recording

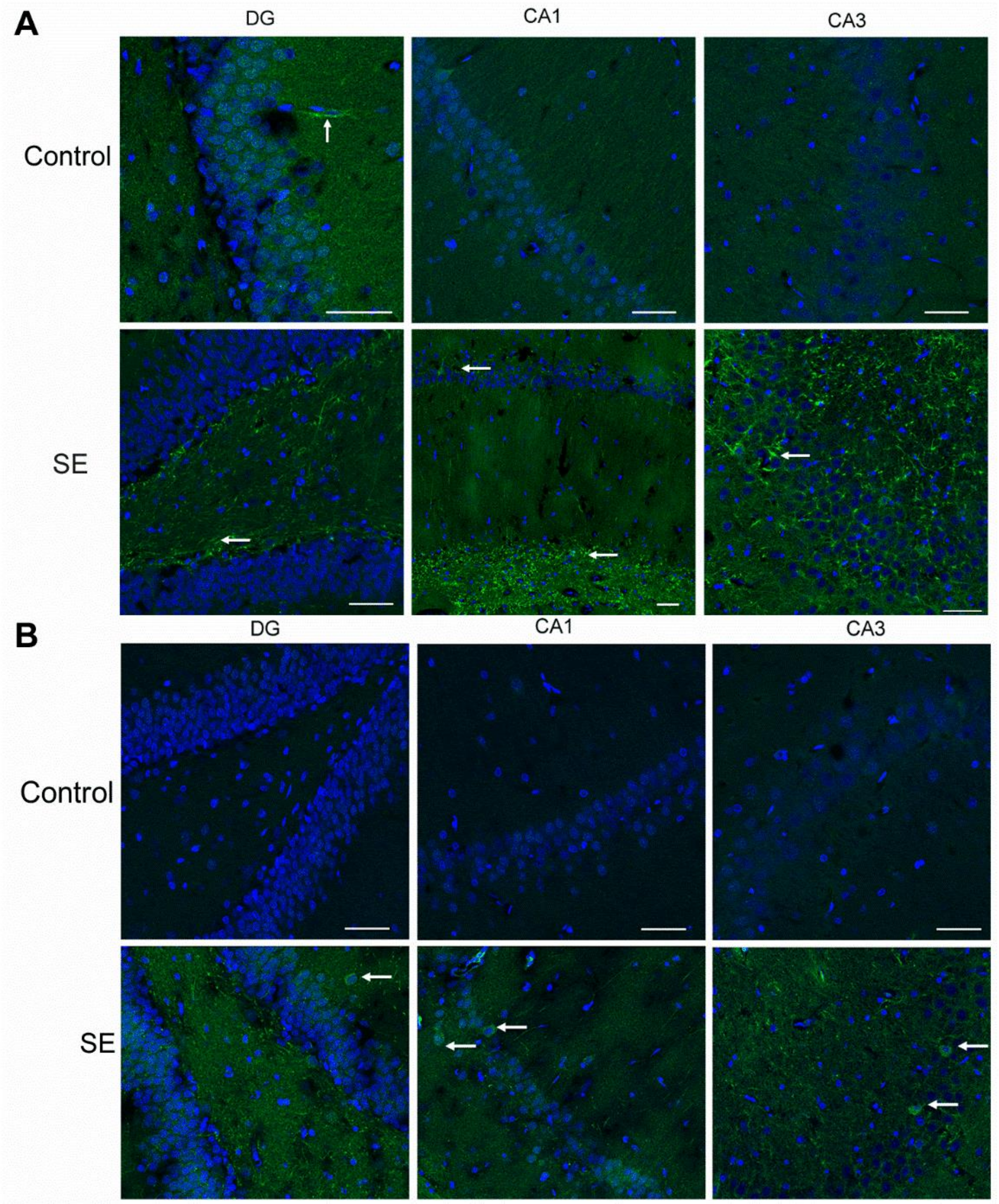

Supplementary Figure 2.VEGF and VEGFR2 expression in the hippocampi of immature rats after SE**(A)** VEGF expression in the hippocampi of immature rats after SE **(B)** VEGFR2 expression in the hippocampi of immature rats after SE (VEGF: green; VEGFR2: green; DAPI: blue; 400×; scale bar: 50 μm).
